# Additive manufacturing: Frameworks for chemical understanding and advancement in vat photopolymerization

**DOI:** 10.1557/s43577-022-00343-0

**Published:** 2022-07-11

**Authors:** Johanna J. Schwartz

**Affiliations:** Lawrence Livermore National Laboratory, Livermore, USA

**Keywords:** Additive manufacturing, Polymer, Photochemical

## Abstract

Three-dimensional printing, or additive manufacturing (AM), is a broad term for a wide range of fabrication methods utilizing materials such as small-molecule, polymer, and metal feedstocks. Each method requires different chemical, physical, and engineering needs to be successful. This article will discuss some of the considerations for polymer-based AM methods. Ultimately, we focus on the chemistries of vat photopolymerization, in which light is used to cure a resin from liquid to solid, to provide an example of how chemical advancements have led to increased speed, resolution, and multimaterial printing capabilities not previously possible.

**Graphical abstract:**

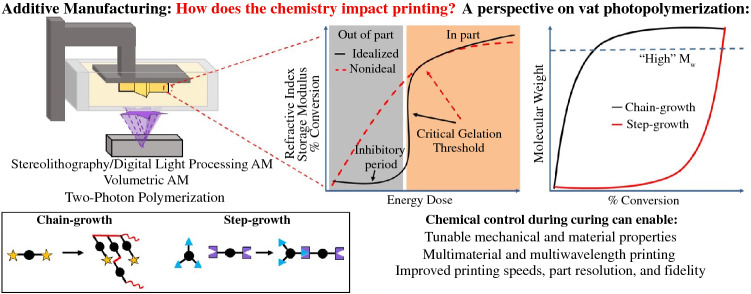

## Introduction to three-dimensional printing

From personalized medicine to aerospace engineering, the design and fabrication freedoms of three-dimensional (3D) printing have led many to call it an industrial revolution.^1^ Three-dimensional printing, also termed additive manufacturing (AM), builds objects by adding material generally in a layer-by-layer fashion, until the final structures are achieved. AM enables users to go directly from a digital model to a 3D object without the need for molds or machining. In addition to geometric freedom, AM approaches can also incorporate multiple materials within a single object and allow users to change materials mid-print to produce more complex blends, with tailored mechanical and design properties, compared with other manufacturing approaches. As there are many different types of AM methods, printable materials, including polymers (or plastics), metals, ceramics, glasses, wood, food, and even living cells are possible.^2^

**Figure 1:**
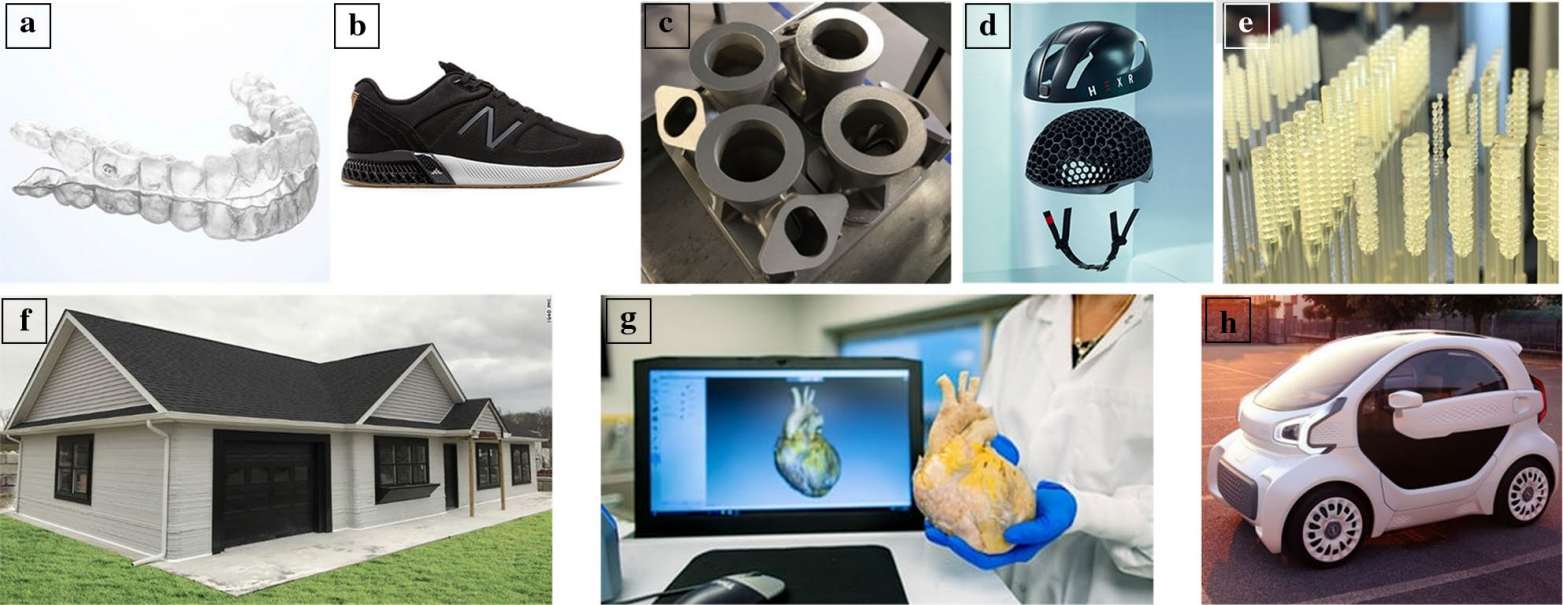
Examples of 3D printing in daily life: (a) Directly printed clear dental aligner without the need of a mold.^8^ (b) New Balance 990 Sport TripleCell sneakers with printed lattice soles.^3^ (c) GE Aviation has switched from casting to metal 3D printing of vent caps for jet engine turbines.^12^ (d) Hexr’s SLS printed lattice helmets are custom-made for each user for improved performance and fit.^7^ (e) Nasal swabs from the Covidswab Github initiative tested at LLNL, providing assistance during a national swab shortage.^17^ (f) The first legal printed cement home in the United States from SQ4D.^13^ (g) A printed surgical heart model recreated from image reconstruction. Used with permission of Mayo Foundation for Medical Education and Research. All rights reserved.^14^ (h) XEV YOYO 3D printed electric car.^11^ All photos used with permission, and all rights reserved.

AM can meet industrial application needs and enhance designs beyond conventional fabrication methods. Three-dimensionally printed consumer goods include shoes and custom in-soles,^3,4^ makeup brushes and personal razors,^5,6^ lightweight sports helmets,^7^ and custom glasses and clear plastic retainers (see examples in **Figure ****1**a**–**h).^8,9^ In the ﻿automotive industry, companies are striving to make fully 3D printed cars, and current examples of mass-produced 3D printed cars are able to reduce costs by reducing the need for intensive tooling.^10,11^ In aerospace and architectural industries, metal- and concrete-based AM methods have cut fabrication costs and sped up production of jet engine components and entire buildings.^12,13^ Personalized medicine and biology has seen expansive advancements, including prostheses and orthotics, surgical mimics, implants, and even vitamins and pharmaceuticals.^14–16^ During the COVID-19 crisis, AM has helped to meet the national and global needs for supplies, including printed face masks, respirators, and nasal test-swabs.^17^ These examples highlight the ways AM is already impacting daily life.

As visible in Figure 1, many industries and applications have benefited from advancements in AM. However, challenges associated with material feedstock advancement and qualification to meet safety and user needs have limited the adoption of AM technologies. Complementing other articles on hardware and software advancements in AM,^2,18–20^ this article will focus on the underlying chemical principles of commercially and industrially relevant polymer-based AM techniques. The section on  “Polymers, handling requirements, and printer technologies” will briefly discuss generalized macroscopic mechanical and physical properties of polymers and their handling requirements within different technologies. The section on  “VP printing and photopolymerization” will discuss the chemistries that enable various photopolymerization print methods to produce cured end-use print structures directly from liquid resin precursors. Photopolymerization chemistries enable light-based VP methods, including two-photon polymerization [TPP, section on “Two-photon polymerization (TPP)”], volumetric additive manufacturing (VAM, section on “Volumetric additive manufacturing”), and multimaterial and multiwavelength print methods (section on “Multimaterial and multiwavelength VP”). Finally, the section on “Conclusions and outlook” will look to the future of AM, including how researchers and developers are removing the barriers of existing systems, and shifting the framework with which we design objects for fabrication.

## Polymers, handling requirements, and printer technologies

Polymeric materials are comprised of repeat units, or monomers, covalently bound together to form chains and/or networks. Differences in the properties of these various materials stem from changes in the chemical structure, size distribution, and relative physical orientation of the monomers in the polymer backbone. Polymers provide an essential foundation for modern life and range from naturally derived (i.e., cellulose, proteins, and DNA) to synthesized (i.e., nylon, polystyrene, and polyethylene).

Polymers are generally characterized by two key factors: their response to heat (thermoplastic or thermoset), and response to mechanical deformation (elastic or plastic).^21^ When heated beyond their glass-transition temperature, *T*_g_, polymeric materials go through reduction in stiffness. As temperature increases, the chains exhibit an increased freedom in mobility, allowing them to disentangle from each other, decreasing viscosity and stiffness. If the polymer chains are linear and not connected to each through chemical linkages, or cross-links, then heating may ultimately lead to melting (*T*_m_, melt transition temperature), which makes the polymer a thermoplastic material. Melting of thermoplastics can enable manufacturing processes such as molding or extrusion. In contrast, thermosets become irreversibly solid or rigid after curing via formation of chemical cross-links. An example of curing is the transition of resins, such as epoxies or dental adhesives, from liquid to solid as polymers within the material cross-link together. The more polymers that are knotted together, the higher the cross-linking density resulting in a network of tightly bound polymeric material. The cross-links in thermoset materials make them more thermally and solvent resistant, and generally thermosets are hard and brittle materials.

**Figure 2:**
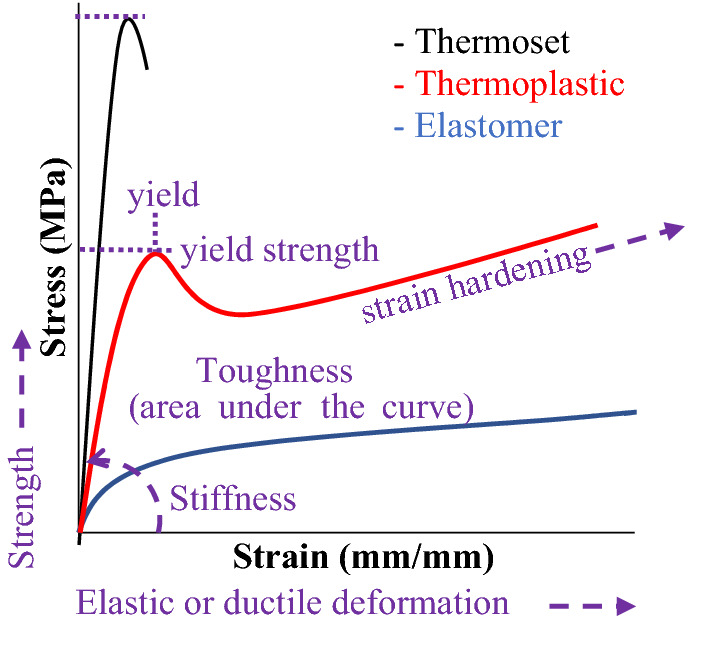
Representative stress–strain curves of tensile elongation of a stiff and brittle thermoset (black), yielding thermoplastic (red), and soft elastomer (blue) materials as well as important macroscopic properties (purple) visible within the data. Elastomers can be thermoplastic or thermoset materials.

Polymer materials can be further characterized by their mechanical responses in terms of deformation (ductile or elastic), strength, stiffness, and toughness (**Figure ****2**). Elasticity refers to the extent that deformations are reversible, in which the material can recover to its original shape. Ductility refers to a material's ability to withstand permanent, or plastic, deformation beyond the elastic zone.^22^ Materials that fully recover their initial shape from elongation without any yielding or plastic deformation, such as rubber, are called elastomers.^22^ Brittle materials are nonelastic and fail at low strains. Under tensile elongation, thermoplastic materials generally exhibit stress–strain profiles containing a recoverable elastic region termed the yield point, after which deformation is permanent (plastic behavior).^23^ The yield stress and maximum stress achieved are also good measures to compare the strength of different materials. Toughness, estimated from the area under the stress–strain curve, is one of the most important parameters for functional object design, though many mechanical and thermal properties are considered when considering a material for a desired application. Factors to consider include examples such as tear strength, impact strength, and abrasion resistance, load deflection, adhesion, thermal aging, and more. Although stress–strain testing is unable to capture every critical factor, it provides a straightforward and quick assessment of material properties for direct comparisons.

**Figure 3:**
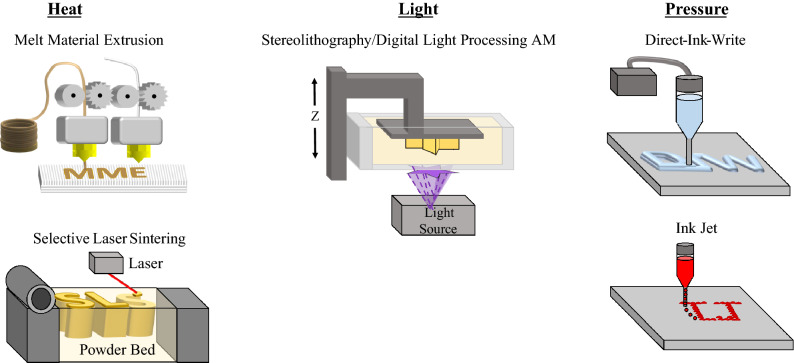
Schematic of five polymer-based additive manufacturing (AM) processes that enable printing through use of heat [melt material extrusion (MME) and selective laser sintering (SLS)], light [vat photopolymerization processes such as stereolithography (SLA, laser light source) and digital light processing AM (DLP AM, projector light source), and pressure (direct ink writing) (DIW, high-viscosity gels) and ink-jetting (IJ, low-viscosity liquids)].

Many types of AM methods exist across the academic, industrial, and general consumer sectors. Methods that have made it to industrial and consumer sectors in general consist of five major AM technologies utilized to print polymers and plastics (as shown in **Figure ****3** and **Table ****I**): fused filament fabrication or melt material extrusion (MME), selective laser sintering (SLS), vat photopolymerization (VP), direct-ink-write (DIW), and ink-jetting (IJ). In these methods, objects are built in a layer-by-layer approach, but each method has its own material input, processing, and handling requirements. MME and SLS processes both utilize heat to melt thermoplastic material into a desired end-use object, either through extrusion (MME) or through laser-induced heating of a powder (SLS). Vat photopolymerization methods, such as stereolithography (SLA) and digital light processing AM (DLP AM) print thermosets and cross-linked elastomers. Finally, DIW and IJ processes use pressure to extrude either filaments of high-viscosity gels (DIW) or droplets of low-viscosity liquids (IJ) into a desired shape. The extruded material can be cured into a cross-linked thermoset using light or heat, or left freestanding as linear thermoplastic layers, depending on the application.

**Table I:** Generalized comparison of different printing methods.

Print Method	Melt Material Extrusion	Selective Laser Sintering	SLA and DLP AM	Direct Ink Writing	Ink Jetting
Entry-system cost ($)^a^	100–1000+	10,000–40,000+	100–1000+	500–5000+	6000–75,000+
Print material	Thermoplastic filaments	Thermoplastic powder	Liquid photoresin	Shear-thinning gel and paste inks	Low-viscosity inks
Material cost ($/kg)	20–40	50–100	50–100	Custom^b^	300–1000
Material curing	No	No	Yes	Sometimes	Yes
Print resolution	100–400 µm	50–100 µm	10–150 µm^c^	100 µm to 2 mm	16–43 µm
Resolution impacts	Nozzle size, layer height^b^	Powder size, layer height,^d^ laser spot size	Pixel/laser spot size, layer height,^d^ curing kinetics	Nozzle size, layer height^d^	Nozzle size
Chemical considerations	∙Modification of filament requires specialized equipment ∙Lack of standardization—batch variability	∙Thermal aging of powder ∙Powder size distribution/batch variability	∙Resin components^e^ ∙Print polymerization kinetics—chemical resolution	∙Ink components^e^ ∙Curing with heat or light possible (ink dependent)	∙Ink components^e^ ∙Curing with heat or light
Physical and engineering considerations	∙Print speed, nozzle size, and temperature effects ∙Overhangs, void spaces difficult	∙Laser intensity and travel speed ∙Bed temperature ∙Powder deposition method/uniformity	∙Low viscosities preferred ∙Light intensity and wavelength impact mechanical properties ∙Optics—*XY* resolution	∙Print speed, nozzle size, and temperature effects ∙Overhangs, void spaces difficult ∙Strict viscosity and shear-thinning rheology requirement	∙Print speed, nozzle size, and temperature effects ∙Overhangs, void spaces difficult ∙Strict low-viscosity ink requirement

^a^Entry-level systems denote relative cost range of the lowest priced consumer systems available. Industrial grade systems may cost more.

^b^Costs estimated from commercially available system formulations. No commercial DIW AM ink companies found as of writing, other than for specific applications (e.g., bioink or silicone printing).^25,26^

^c^Submicrometer resolutions are possible, resolutions reported for entry-level consumer SLA and DLP AM systems.

^d^Layer height resolutions (*Z*-axis) of 50–100 µm common. Print resolutions reported above are planar (*XY*) resolutions.

^e^Chemical components of resins and inks can include the monomers, polymers, solvents, additives, and cross-linkers.

Each AM method has its own material, engineering, and software advantages, considerations, and challenges that ultimately impact the properties of end-use parts and applications. Print considerations also include more practical factors, such as cost-to-entry, material availability, and material processing requirements. General considerations are summarized in Table I. In many cases, material compromises and synthetic modifications to polymers are made to meet the “printability” needs of a particular AM method. For example, a common approach to making well-characterized materials like silicones printable in photo-based AM methods like SLA and DLP AM is to functionalize them with photo-reactive groups such as (meth)acrylates.^24^ Acrylate groups can compromise the downstream material properties of the end-use objects, such as inducing lower mechanical strength and photo-induced aging effects compared to the base polymeric material. At the same time, the benefits of being able to print complex geometries and maintain some of the desired material properties, for example good biocompatibility and thermal insulation, make these compromises worth it for targeted applications. The interplay between physical and chemical considerations in each AM method is a fundamental design parameter that dictates which method is best for a target application.

Practical engineering challenges not only dictate the speed, resolution, and ease-of-use of an AM method, but also the breadth and types of accessible materials.^18^ For example, the upper limit of heating temperature of a MME extrusion system fundamentally limits the choice of thermoplastic materials. Materials such as poly(ether ether ketone) (PEEK), which have a high melt temperature, require a specialized high-temperature extrusion nozzle to be processable.^27^ Materials with higher melt temperatures, such as Nylon, may be more accessible to SLS-based printing methods, but must be processed into powders with uniform size distributions for good printing.^28^ Furthermore, the polymer powder may not be reusable between prints because of thermal processing, producing more waste. The major engineering challenges of processes such as DIW and IJ center around viscosity limitations that impact their material scope. Additionally, gels and inks used in DIW and IJ methods often need secondary curing chemistries (such as the photochemistries discussed in the next section on “VP printing and photopolymerization”) to enable long-term chemical and wear-resistance. The interplay between engineering and chemistry varies based on the printing method, creating a framework for identifying which AM method to choose for each desired material. Of the different AM methods, VP has one of widest ranges of accessible materials and chemistries. Although viscosity limitations still need to be considered, the range of tolerable viscosities is wider for VP processes. Additionally, in VP the polymerization chemistries happen directly during the printing process, making the engineering and chemical considerations highly interrelated. This article will highlight how understanding the chemical framework of an AM method such as VP can lead to innovation and advancement.

## VP printing and photopolymerization

**Figure 4:**
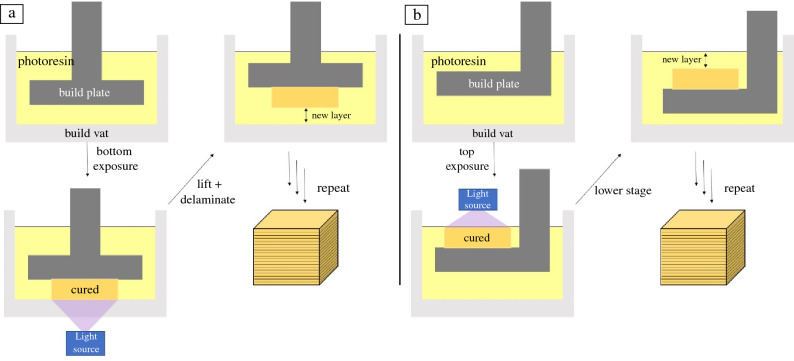
Schematic of layer-based vat photopolymerization printing process. An image from the laser (stereolithography) or projector (digital light processing additive manufacturing) is exposed (a) through the bottom of the build vat, or (b) from the top of resin vat, curing the liquid photoresin, the *z*-stage build plate lifts or lowers, filling in liquid under or over the cured layer, respectively. Another layer is exposed, and the process is repeated to build the object.

VP is a generalized term that covers a large range of different photo-based AM methods, including SLA, DLP AM, TPP, and VAM. SLA and DLP AM both build objects in a vat of liquid photoresin by curing in a layer-by-layer fashion (**Figures** 3 and  **4**), and can make macroscale objects with microscale features.^29^ SLA uses laser scanning to irradiate and cure each layer, whereas DLP AM uses a digital micromirror array to cure a patterned layer all-at-once. SLA and DLP AM resins generally rely on acrylate-based free-radical chain-growth polymerizations. The small-molecule monomers of the resin are light- and heat-sensitive, and in some cases can have a high volatile content requiring the use of fume hoods or well-ventilated spaces for printing in some instances. In both SLA and DLP AM, the orientation of the build platform relative to the light source impacts the materials accessible for fabrication (top-down irradiation, or bottom-up). In Figure 3, the SLA/DLP AM setup is in a bottom-up configuration, meaning that each layer of photoresin is cured between a transparent bottom surface (such as a glass or quartz container) and the build platform. After a layer is cured, the build platform needs to move up to delaminate the printed object and let more resin infill for the next cure slice. If the resin is high-viscosity, or too adhesive, the delamination and recoating step can lead to part failure. By simply moving to a top-down configuration (Figure 4b, where the light displays from above, and the build platform moves down into the resin vat to make each layer) issues of adhesion can be mostly mitigated; however, high-viscosity resins may still need other engineering solutions for effective recoating. Additionally, in a top-down approach, the main curing layer is exposed to air. Many VP photochemistries (such as free-radical polymerization of acrylates), as will be discussed next, are oxygen-sensitive, which could impact the degree of curing. Understanding the polymerization chemistries involved can enable modifications to the VP machine to mitigate concerns.

**Figure 5:**
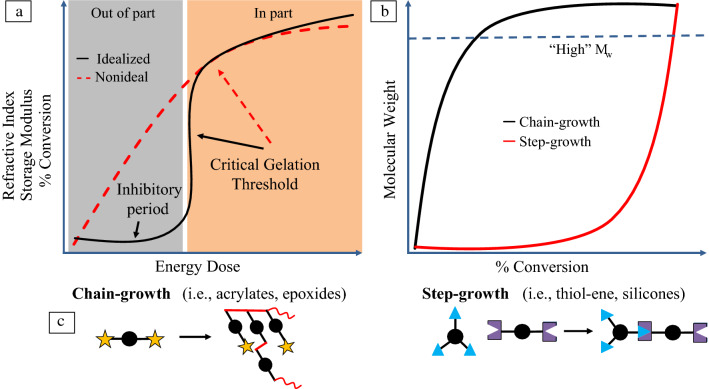
(a) Idealized kinetic profile for vat photopolymerizations. Gelation and solidification should only occur in desired pixels or voxels (in-part) and not in print void spaces (out-of-part). Energy dose relates to the light energy accumulated during a print including the intensity of irradiated light, resin absorbance, and the time of irradiation. Lack of inhibitory period in either chain-growth or step-growth polymerizations (red dashed line) could lead to a decrease in-part resolution and fidelity. (b) Polymer molecular weight profile relative to the percent conversion of resin monomer. Chain-growth polymerizations achieve high molecular weight and gelation (blue dashed line) at low percent conversion of monomer. (c) Simplified schematic representation of chain-growth and step-growth polymerizations. Red lines in chain-growth denote new covalent bonds from star end functional groups. Center ball denotes polymer backbone. Step-growth polymerizations require two different (blue triangle and purple rectangle) multifunctional monomers to react together to create a cross-linked polymer network.

Polymerization chemistries are generally categorized by the mechanism in which monomeric repeat units combine and grow the polymer material: step-growth or chain-growth. Step-growth polymerizations involve the stepwise reaction of multifunctional monomers to eventually form large polymer chains one block at a time. Chain-growth polymerizations involve the production of “active sites” that monomers react with, producing growing polymer with an active chain end.^21^ It is important to note that when discussing VP chemistries and materials, in general it is for curing thermosets. The polymerization mechanism used can thereby dictate downstream macroscopic properties of the printed thermoset. Many current commercial photopolymer resins in VP systems use free-radical-mediated chain-growth polymerization chemistries of materials such as acrylates.^30^ This is because chain-growth polymerizations can achieve long-chain polymers, which will ultimately cure and solidify within the resin, with a relatively low percent of monomer conversion (**Figure ****5**). Photopolymerization of step-growth reactions are less common and not often used in VP processes as it has issues with stability and kinetic control and requires high conversion of the reactive monomers to reach solidification.

**Scheme 1:**
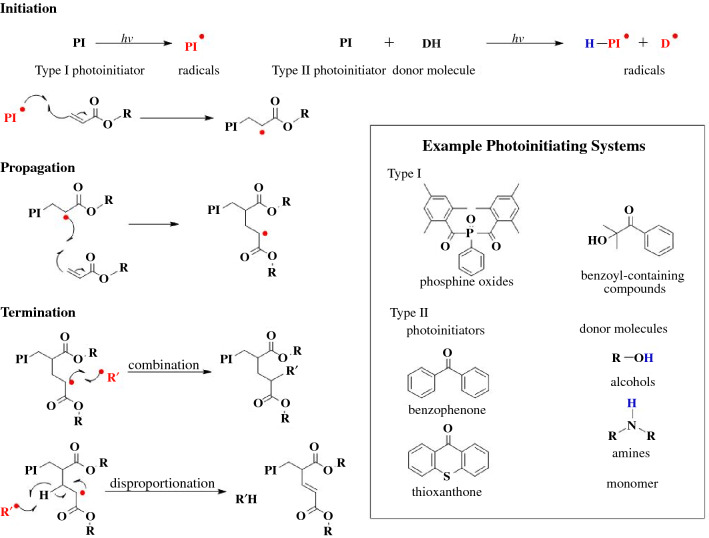
Initiation, propagation, and termination mechanisms for free-radical-mediated chain-growth of acrylates. Radicals (red) and donor hydrogens (blue) highlighted. For photoinitiation, example Norrish Type I and Norrish Type II photoinitiators are presented.

There are three important mechanisms that occur during chain-growth polymerizations: initiation, propagation, and termination (**Scheme ****1**). In the case of VP chemistries, we will focus in on free-radical initiated chain-growth polymerizations. Initiation indicates the start of polymerization in which first a compound responds to light, producing a radical, followed by the reactive radical species interacting with a monomer.^31^ Propagation is the continued reaction of the reactive species with more monomer. Termination is any process that ends the growth of a chain, and generally occurs through either combination or disproportionation. Combination is where two active chains couple together, forming one nonreactive chain. Disproportionation involves hydrogen abstraction from one active chain end to another, resulting in two polymers, one with a saturated chain end and an unsaturated chain.^32^

At a critical monomer conversion percentage, the polymer will transition from a liquid to a solid, curing the material. This transition is known as gelation, and it is the main chemical parameter for control of object resolution. Chain-growth polymerizations achieve gelation at a relatively low conversion (Figure 5), making it easy to form the object with relatively low curing. After printing, the structure can be cleaned and post-cured for increased conversion and strengthening of end-use object. The tradeoff for early gelation is that the polymer network formed in cross-linkable chain-growth polymerizations is often nonuniform, experiencing regions of high and low-cross-linking that impact the final mechanical properties of the printed part. Network nonuniformity often makes chain-growth acrylate materials brittle.

Generally in free-radical photopolymerizations, irradiation with ultraviolet or visible light results in either direct cleavage of the photoinitiator to produce a radical or photoexcitation of the photoinitiator, which then abstracts an electron or hydrogen from a donor molecule to produce a radical. Photoinitiators that cleave directly to produce a radical are called Norrish Type I initiators and are generally what is used in VP.^30,33^ Photoinitiators that need a secondary, often intermolecular, reaction step to produce a radical are called Norrish Type II initiators. Because of the intermolecular interactions needed for Norrish Type II initiators, the resin viscosity, initiator concentration, and ambient temperature all impact the rate of initiation, limiting their use in VP processes.

**Scheme 2:**
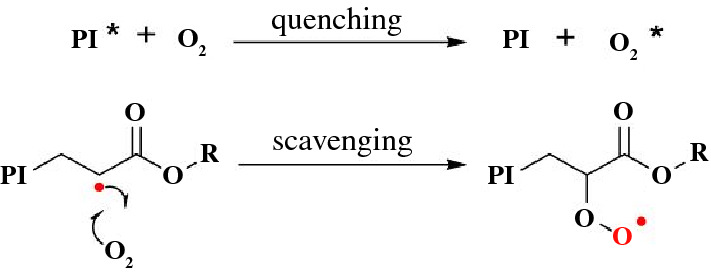
Oxygen inhibition of photoinitiator (PI) and polymer free-radical polymerization of acrylates. Oxygen can quench the triplet state of reactive PI (PI*). Radicals highlighted as red.

Differences in light intensity, photoinitiator absorption, and photoinitiator efficiency or quantum yield alter the amount of active radicals in a polymerization, effecting both the initiation and propagation steps of a polymerization.^34^ Temperature differences vary the rate of propagation. For example, high temperatures or exothermic reactions can potentially result in decomposition of the initiator, which can produce more radicals, or even autocatalyze the thermal polymerization of the monomers themselves.^35^ A major issue in VP of free-radical acrylate resins is oxygen inhibition, which is capable of quenching the triplet state of the reactive photoinitiator and stopping the production of a reactive radical.^36^ It can also react with the propagating chain end carbon-centered radical, producing a less reactive oxygen-centered peroxy radical that does not react with monomer (**Scheme ****2**).

In VP, many methods are used to minimize the environmental and system impacts on the resin photopolymerizations. Although light intensity and temperature impacts can be mitigated through engineering, software, and climate controls, oxygen inhibition in free-radical polymerizations is more difficult to mediate. Luckily, oxygen permeability is low in acrylate resins, with the top 5 µm of resin being most impacted.^37^ By printing from the bottom of the vat (Figure 4a) of liquid photoresin, the dissolved molecular oxygen can be consumed. Increased concentrations of photoinitiator reduces the impact of inhibition. Finally, oxygen solubility is resin dependent, as some free-radical monomers are more oxygen-sensitize than others. With a bottom-up printing approach (irradiating light up from the bottom of the build vat), increased concentrations of initiator, and judicious monomer choices, oxygen inhibition can be mostly mitigated. Acrylate monomers are easy and cost-effective to functionalize, synthesize, and use, which has led to many commercial acrylate-based materials and resin formulations.

Although printing from the bottom of the vat can mitigate issues with oxygen, as mentioned earlier it provides an added constraint of layer delamination. If the object adheres too strongly to the bottom surface, the layer can delaminate from the build plate, causing the print to fail. Additionally, the delamination step adds time to the print process. One solution is to make the bottom surface a liquid instead of solid, using dense fluids that are immiscible with resin.^38^ Another interesting solution to the delamination problem is to add oxygen back into the resin at that bottom surface, creating an inhibition window through an oxygen permeable membrane, so that curing is inhibited and the object does not stick. This process is known as continuous liquid interface production (CLIP) DLP AM.^39^ Carbon 3D utilizes this in their printers to speed up printing, by removing the delamination step of standard VP layer-by-layer assembly. Instead, they continuously grow the part out of the vat of resin as the build plate is elevated.^40^ In essence, an inherent property of free-radical polymerizations that is generally considered a limitation was instead harnessed as a benefit.

**Scheme 3:**
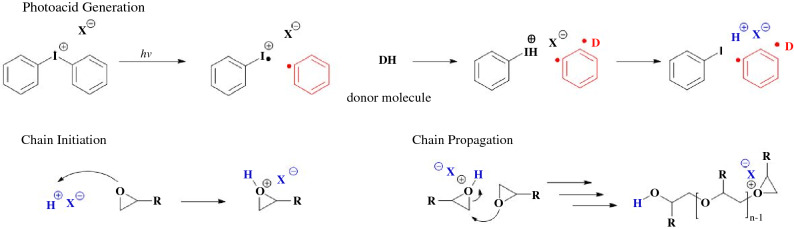
Generalized mechanism of chain-growth-mediated cationic epoxide polymerization. Proton donor molecules include examples such as solvent and resin. In the process of generating acid (blue) for chain initiation, photoacid generators like diphenyliodonium salts produce two radical byproducts (red).

Chain-growth polymerization mechanisms that do not utilize free radicals, such as cationic polymerizations, provide alternative material options.^41^ Cationic polymerizations utilize the generation of acid (protons (H^+^) to facilitate polymerization instead of free radicals (**Scheme ****3**). Cationic polymerizations, in particular cationic ring opening polymerizations of monomers such as epoxides, are already used in VP, and are desired for their higher strength and toughnesses compared to acrylate-based materials.^30,42^ Epoxides also exhibit good layer adhesion and less shrinkage during polymerization than acrylate materials, which is beneficial for print fidelity. Epoxide polymerizations are generally slower than acrylate polymerizations, as propagation of the heavier acid or proton molecule within the resin is slower than a small, unstable radical.^43^ Cationic polymerizations are also generally living in character. This means the reaction would continue without termination until all monomer is consumed or is diffusion-limited (the cured object becomes solid, and chains are no longer mobile). In practice this is more complex, as the epoxide heterocycles are susceptible to nucleophilic attack by other electron-rich species. With photoresins that have mixtures of many different chemical components, nucleophilic attack may be possible, but the slow conversion of cationic polymerizations is still its largest limitation preventing more widespread use in VP processes.

**Scheme 4:**
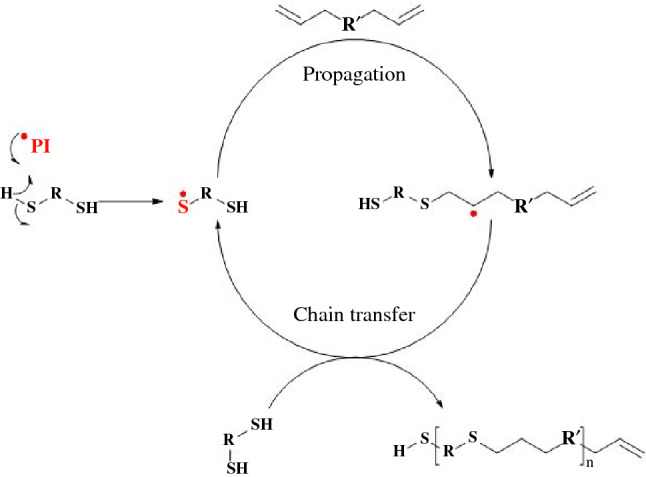
Photo-mediated step-growth polymerization of difunctional thiol-ene monomers. Unlike chain-growth, difunctional monomers would result in a linear polymer. Higher order multifunctional monomers are needed for thermosets. Radicals are highlighted as red.

Step-growth polymerizations have a very different conversion profile than chain-growth. Polymer is slowly built up from the reaction of multifunctional monomers bit-by-bit, and only at high conversions is gelation achieved (**Scheme ****4**). In photoinitiated chain-growth polymerizations, the number of activated photoinitiators dictates the number of growing chains, and by extension the overall polymer network structure during curing. The growing chains after initiated, exist throughout the propagation steps until terminated. In photo-mediated step-growth polymerizations, photo-mediators or photocatalysts activate a single condensation or addition of two reactive groups. The radical does not propagate along to grow a chain; instead the radical can undergo chain transfer to induce another addition or condensation with other reactive groups within the resin. During VP, radicals are produced where light is present. Gelation and solidification are utilized to limit diffusion and maintain print resolution, similar to cationic polymerizations.^44^ As gelation occurs later, the network formed from step-growth polymerizations is more uniform.^45^ Objects formed through step-growth polymerizations thereby have improved mechanical properties compared to similar compositions achieved through chain-growth polymerizations.^46^ For example, thiol-ene step-growth polymerizations have been investigated within the VP field, and have shown tunable mechanical properties and improved toughness relative to similar acrylate-based resin systems.^46^ Expanding on this initial work, Schwartz and co-workers found the thiol-ene photoresins produce printed structures with stimuli-responsive shape-memory behavior.^47^ Ultimately, coupling the wider range of chemical synthetic freedom, network uniformity, oxygen insensitivity, and reduced shrinkage of step-growth polymerizations, researchers have begun to target step-growth polymerization systems for expansion of VP chemistries.

In addition to improved toughnesses and network uniformity, VP of step-growth materials could also produce thermosets with degradable or dynamic bonds for downstream reprocessing.^48–50^ Step-growth polymerizations could make thermoset materials with similar processability to thermoplastics, but maintained mechanical and wear resistance. Non-permanent cross-links could enable tunable mechanical properties, self-healing, and recyclability. The interplay between thermoplastic and thermoset is heavily investigated in the field of polymer chemistry, but has only recently been incorporated into AM.^51^ For example, Bowman and co-workers have utilized dynamic photoprintable thiol-thioester and thiol-ene anhydride networks to create vat photopolymerized thermally reconfigurable and degradable thermoset structures.^48,52^ Expanding VP formulations to include other photo-mediated step-growth and dynamic covalent chemistries, such as Diels–Alder reactions, amide and ester chemistries, would give VP structures the ability to simultaneously have improved toughness and wear resistance as well as potential self-healing and stimuli-responsive behavior, reprocessability, and recyclability.^53,54^

One way that researchers have sought to overcome limitations of different resin chemistries for VP is to utilize mixed resin systems that incorporate multiple polymerization chemistries. Dual-cure networks are an example of this, in which two orthogonal polymerizations occur within the same structure. This creates an interpenetrating network of two different polymers, often having improved mechanical properties compared to their homopolymerized counterparts.^55^ In VP, dual-cure networks often use mixed photoresins containing acrylates and epoxides to circumvent the slow polymerization of epoxides. The acrylate resin component can be photopolymerized quickly to increase print speed and achieve shape fixity, and the epoxide resin component can be further photo- or thermally polymerized to improve material properties and chemical resistance.^56^ This has also been shown to be beneficial in mitigating the issues of both radical and cationic polymerizations.^43,57^ Incorporation of epoxide monomers in acrylate resins can decrease the oxygen inhibition of the free-radical polymerization.^57^ Similarly, acrylate monomers can decrease the moisture sensitivity of cationic polymerizations.^57^ These more complex hybrid formulations come with other issues, such as phase separation and shrinkage differences, but they also provide an example of how knowledge of chemical formulations and polymerization mechanisms can help provide a means to move beyond the brittle materials commercially available. For VP processes to progress toward engineering and functional applications, materials with improved properties, and systems that mitigate the chemistry limitations of the polymerizations, are necessary.

## Two-photon polymerization (TPP)

**Figure 6:**
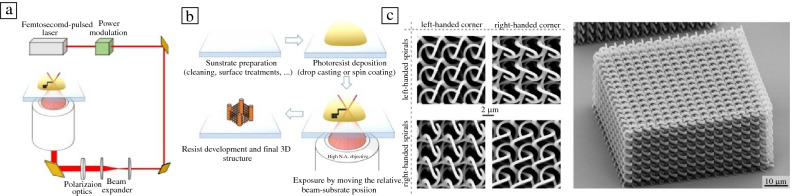
Schematic of direct laser writing two-photon polymerization (DLW TPP) with (a) simplified optical path of TPP system and (b) simplified procedure of 3D sample fabrication. Reused with permission.^66^ (c) Example of scanning electron microscope images of DLW printed bi-chiral photonic crystal structures with submicrometer features.^67^ Images reused with permission.

To achieve objects with submicron resolutions, two-photon polymerization (TPP) AM methods are commonly used, such as direct laser writing (DLW) (**Figure ****6**). In DLW TPP, a pulsed laser is focused into a volume of photosensitive resin. Pulsed lasers are used to generate sufficiently high intensities (TW/cm^2^) to allow for multiphoton absorptive processes.^58^ At the heart of this process is the simultaneous absorption of two or more photons (TPA) from the laser in a single event by a photosensitive molecule, which in turn, initiates local polymerization chemistries, such as those highlighted in the section “VP printing and photopolymerization.” In contrast to one-photon absorption of previously described VP methods, TPA is a nonlinear process in which the probability of TPA occurring is related to the square of the photon flux density. Because DLW TPP relies on the simultaneous absorption of photons within the same volume, features can be smaller than the diffraction limit of the optical system. TPP generates objects through moving the laser focal point through the resin volume in all three dimensions, with feature sizes as small as 100 nm possible.^59^ Submicrometer resolutions are achieved through optimization of the optical and engineering parameters such as light intensity, laser wavelength, pulse duration, scan speeds, and focusing lens objective.^60,61^ Additionally, modulating the chemical and physical properties of the photoresin, such as refractive index, can further improve the resolution of each discrete volume element, or voxel, and enable TPP printing of tall (>1 cm) structures.^62^ While high-resolution objects are possible, conventional TPP print speeds are very slow, around 0.1 mm^3^ per h, meaning an object as large as a cubic centimeter could take days to weeks to print. Through modifications of the laser pulsing and optical systems, researchers have been able to speed up TPP printing processes, to as fast as 10–100 mm^3^ per h, reducing print costs indirectly by maximizing the laser lifetime.^63–65^

Many process parameters go into facilitating good resolution in lithographic and TPP fabrication, and yet the underlying chemistries of TPP processes are largely the same as microscale and macroscale VP methods. This creates a generalized ability to understand chemical resolution within systems through control and analysis of bulk photochemical conversion. As discussed in the section “VP printing and photopolymerization” most resins used in VP to date have centered on free-radical chain-growth polymerization of acrylates, which is largely true of TPP and lithographic approaches as well.

## Volumetric additive manufacturing

**Figure 7:**
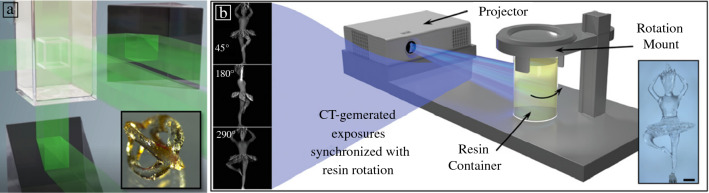
(a) Holographic volumetric additive manufacturing (VAM)—uses overlap of perpendicular projections. (b) Tomographic VAM—uses projection of computed tomography (CT)-generated exposures into a rotating resin vat. Images reused with permission.^68,69^

Volumetric additive manufacturing (VAM) is an emerging paradigm in 3D printing that extends the conceptual frameworks of other VP approaches. Whereas layerwise methods such as SLA and DLP AM produce objects by subdividing them into low-dimensional subunits (0D voxels, 1D lines, or 2D layer slices), VAM generates an entire 3D object all-at-once, without layers. VAM methods rely on controlling the accumulation of light energy absorbed at a voxel within a photosensitive resin vat. When sufficient light energy, or volumetric energy dose, is reached within a voxel, curing and solidification occur (**Figure ****7**).^68,69^ Several VAM techniques have been demonstrated, including multibeam holographic and reverse-tomographic methods, which implement spatial and temporal accumulation of the 3D energy dose, respectively. Holographic VAM uses the simultaneous overlap of multiple patterned light fields to accumulate dose during printing in a stationary resin container.^68^ Tomographic VAM converts a 3D model into angular projections using algorithms derived from computed tomography, and the angular images are projected sequentially into a rotating vat of resin.^69^ VAM offers a range of promising opportunities for material design and performance in the context of AM. Because VAM printing is layerless, isotropic structures without layering effects become possible (unlike SLA or DLP AM in which layering can impart mechanical anisotropy as layers can delaminate). VAM thereby can create structures whose mechanical and physical properties are similar to their bulk-cured or molded counterparts.^46^ Additionally, because the entire object is cured in a single step, the speed of printing is much faster than traditional VP methods, lasting only 10–100 s.^46^ Whereas other VP methods are often limited to low-viscosity resins, VAM can utilize high-viscosity materials, expanding material versatility.

Control over the chemistries discussed in the VP section becomes even more critical in VAM, as bulk curing and solidification kinetics directly control the resolution of the single-step 3D part (Figure 5). During the VAM process the entire build volume is irradiated with light, but the patterned projected images are generated such that only the in-part voxels have enough accumulated volumetric energy dose to reach gelation. Ultimately, detailed characterization of the relationship between absorbed optical energy, chemical conversion, and material mechanical properties is required to obtain the desired final structure. Underestimating the cure dose will result in unformed or incomplete objects, and overestimating dose will mean that out-of-part voxels may reach gelation, creating outgrowth. With these considerations in mind, researchers have developed methods to print both chain- and step-growth polymerization-based resin materials, and even cell-loaded hydrogels, with VAM.^70^ Important efforts needed in continued research will be to attain fine control over polymer network morphology on the nano-, micro- and macroscale levels. This will require tuning print parameters, such as light intensity and time of print, and incorporating computational simulations tools to study local material response.^71^ Furthermore, incorporation of multiwavelength capabilities with VAM provides an exciting opportunity for complex multimaterial and high-resolution structure fabrication.^72,73^

## Multimaterial and multiwavelength VP

In nature, objects and biological structures are rarely if ever made entirely of one single material. One of the natural benefits of AM technologies is the ability to generate multimaterial structures within a single fabrication step.^74^ For example, MME uses both multi-head extrusion and mixing nozzles to create heterogenous and blended multimaterial parts (Figure 3).^74,75^ Similarly, IJ and DIW inherently have multimaterial capabilities, limited only by the number of ink deposition nozzles accessible within the hardware of the machine. DIW and IJ have been used to make complex multimaterial parts for applications, including bioprinting of organs and soft matter, soft robotics, and making mechanoresponsive load-bearing structures.^75–77^ The hardware and design of nozzle-based AM methods inherently enable multimaterial fabrication, whereas methods such as SLS and VP can achieve multimaterial structures through simple engineering and print changes, such as swapping out the powder or resin during printing.^38^ Swapping material is time-intensive, and limits multimaterial structures to stripes or layers of different materials.^38,78^

**Figure 8:**
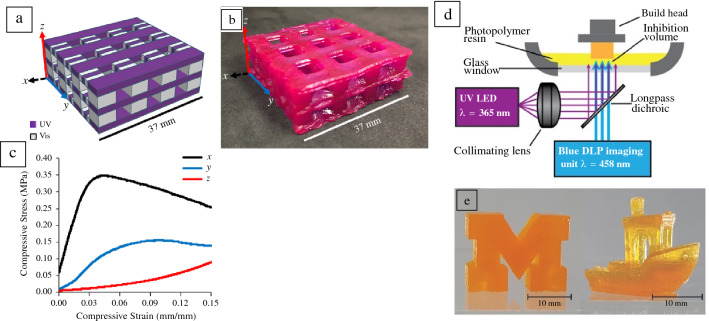
Multimaterial actinic spatial control vat polymerization (VP) (a) design (purple, UV cured; white, visible light cured) and (b) printed lattice structure with (c) anisotropic mechanical properties in all axes of printing from hard (UV cured) and soft (visible light cured) domains. Reused with permission.^81^ (d) Optical setup for two-color VP by concurrent photopolymerization and photoinhibition. (e) Solid block M (left) and tug boat (right) printed using the two-color photopolymerization/photoinhibition stereolithography system at 500 and 375 mm/h, respectively. Reused with permission.^83^

Increasing the chemical complexity and control of the materials used in the AM method (such as the resin of VP systems) could facilitate improved multimaterial control. For example, simple changes to light intensity can modulate the initiation and propagation kinetics in free-radical acrylate systems during VP, which can affect the material network structure and, in turn, overall mechanical properties in printed structures. This means that changing to grayscale printing could create graded and heterogeneous structures with complex mechanical responses.^79,80^ Taking it a step further, Boydston and co-workers use mixed acrylate and epoxide resin systems to produce multimaterial objects through a novel multiwavelength DLP AM approach called multimaterial actinic spatial control (MASC) VP.^81^ Through simple changes to irradiation color during printing, they could selectively polymerize a free-radical acrylate system or both acrylates and cationic epoxides in tandem. A soft, hydrophilic acrylate and a hard, hydrophobic epoxide material were chosen such that the multimaterial objects had over three orders of magnitude differences in stiffness between their soft and hard domains (**Figure ****8**a**–**c). Swelling differences within the multimaterial parts were also used to create actuating bioinspired sea star structures. The MASC VP approach highlights the benefits of using controlled chemistries to increase print complexity from the same vat of resin. With further expansion beyond acrylate homopolymerizations, and further control over the kinetics and degree of polymerization in VP systems, compositional complexity continues to grow.^82^

Multiwavelength additive manufacturing systems have also been used to increase resolution in printed parts through photoinhibitory chemistries (Figure 8d–e).^72,83^ One wavelength is used to initiate polymerization, often free-radical-mediated acrylate polymerization, and another to produce radical inhibitors that trap and stop propagating radicals. Photoinhibition limits the impact of outgrowth and can be used to maintain hollow geometries that may otherwise lose resolution. Similar to Carbon3D’s CLIP approach mentioned in the section on “VP printing and photopolymerization,” photoinhibitory approaches were also used to create a continuous SLA print method.^83^ An inhibition window, or an area with a high concentration of inhibitors, was generated in a bottom-up SLA system through UV irradiation directly at the curing-irradiation plane. The inhibition window prevents the cured layer from sticking to the bottom build vat surface, enabling Scott and co-workers to continuously raise their cured print out of the resin without layering.^83^ Dual-wavelength multimaterial and photoinhibitory examples highlight the potential of VP methods with increased photochemical toolboxes. Although highlighted for SLA and DLP AM methods, complex multimaterial and photoinhibitory approaches have been used in TPP and VAM VP methods as well.^72,73,84^ In the future, the entire spectrum of light could become amenable for new levels of compositional control in end-use objects.

## Conclusions and outlook

AM has already become the state of the art in fields such as aerospace (engine and ducting components), medicine (prostheses, organs-on-a-chip, dental, and surgical modeling), transportation, energy, and consumer devices. AM technologies have critical advantages with their unique multimaterial and prototyping capabilities. These capabilities have been enabled by inherent hardware and engineering advancements, such as utilizing multiple extrusion nozzles in MME, DIW, and IJ processes or exchanging the resin or powder during printing in DLP AM, SLA, and SLS techniques. In the case of DLP AM and SLA, while it is simple to swap resins mid-print to create multimaterial structures, it significantly increases the time of printing and multimaterial objects are limited in complexity. Increased control and complexity within VP cure chemistries and material feedstocks can advance the print method beyond what is possible from hardware modifications. Although this article focused mostly on VP processes, understanding the chemical frameworks of other AM methods could lead to further innovations and increased compositional complexity in end-use objects.

For AM to expand to other large-scale industrial uses and global production applications, a wide range of cost-effective materials with diverse mechanical and physical properties need to be developed. In addition, the printing process itself needs to have high precision, resolution, and speed. In this article, we highlighted the potential of using VP chemistries to increase compositional and production control of end-use objects. Currently, VP systems generally rely on acrylate-based photoresins. By expanding the materials accessible to VP (such as moving to dual-cure and step-growth polymerizations) users can mitigate many limitations of acrylate polymerizations, such as oxygen sensitivity, and achieve structures with modular and tunable mechanical properties, and stimuli-responsive and dynamic behavior. Examples in this article include utilizing step-growth thiol-ene chemistries to produce end-use objects with reconfigurable and degradable bonds, and thermal shape memory. Chemical limitations that can negatively impact VP chemistries, such as oxygen inhibition of free-radical acrylate polymerizations, can also provide areas for innovation, such as the development of high-speed CLIP printing using an inhibition window at the build plate. Simple changes in irradiation parameters, such as light intensity and wavelength, increase control over network formation to produce heterogeneous, graded, and multimaterial structures. Multiwavelength VP systems enable complex and mixed photoresin formulations for multimaterial and photoinhibitory chemistries. Different wavelength projections can be used to generate multimaterial objects with large differences in mechanical properties (more than three orders of magnitude variation in stiffness produced from mixed acrylate epoxide photoresins), structures with improved part fidelity, and overall faster print times. So far, only two material and dual wavelength resins have been reported. Expanding to three, four, and beyond can create objects physically impossible to produce through other manufacturing methods. Increased chemical freedom can only make the use of 3D printing more widespread.
